# Form-Vessel Classification of Cholangioscopy Findings to Diagnose Biliary Tract Carcinoma’s Superficial Spread

**DOI:** 10.3390/ijms21093311

**Published:** 2020-05-07

**Authors:** Yoshimitsu Fukasawa, Shinichi Takano, Mitsuharu Fukasawa, Shinya Maekawa, Makoto Kadokura, Hiroko Shindo, Ei Takahashi, Sumio Hirose, Satoshi Kawakami, Hiroshi Hayakawa, Tatsuya Yamaguchi, Yasuhiro Nakayama, Taisuke Inoue, Tadashi Sato, Nobuyuki Enomoto

**Affiliations:** First Department of Internal Medicine, Faculty of Medicine, University of Yamanashi, Chuo, Yamanashi 409-3898, Japan; ii258pp2@yahoo.co.jp (Y.F.); fmitsu@yamanashi.ac.jp (M.F.); maekawa@yamanashi.ac.jp (S.M.); makotok@yamanashi.ac.jp (M.K.); shindoh@yamanashi.ac.jp (H.S.); etakahashi@yamanashi.ac.jp (E.T.); sh99073@yahoo.co.jp (S.H.); k234_0516@yahoo.co.jp (S.K.); hhayakawa@yamanashi.ac.jp (H.H.); ytatsuya@yamanashi.ac.jp (T.Y.); ynakayama@yamanashi.ac.jp (Y.N.); tinoue@yamanashi.ac.jp (T.I.); tadashis@yamanashi.ac.jp (T.S.); enomoto@yamanashi.ac.jp (N.E.)

**Keywords:** bile duct cancer, cholangioscope, genetic mutation, tumor spread, biopsy

## Abstract

We aimed to evaluate a newly developed peroral cholangioscopy (POCS) classification system by comparing classified lesions with histological and genetic findings. We analyzed 30 biopsied specimens from 11 patients with biliary tract cancer (BTC) who underwent POCS. An original classification of POCS findings was made based on the biliary surface’s form (F factor, 4 grades) and vessel structure (V-factor, 3 grades). Findings were then compared with those of corresponding biopsy specimens analyzed histologically and by next-generation sequencing to identify somatic mutations. In addition, the histology of postoperative surgical stumps and preoperative POCS findings were compared. Histological malignancy rate in biopsied specimens increased with increasing F- and V-factor scores (F1, 0%; F1, 25%; F3, 50%; F4, 62.5%; *p* = 0.0015; V1, 0%; V2, 20%; V3, 70%; *p* < 0.001). Furthermore, we observed a statistically significant increase of the mutant allele frequency of mutated genes with increasing F- and V-factor scores (F factor, *p* = 0.0050; V-factor, *p* < 0.001). All surgical stumps were accurately diagnosed using POCS findings. The F–V classification of POCS findings is both histologically and genetically valid and will contribute to the methods of diagnosing the superficial spread of BTC tumors.

## 1. Introduction

Biliary tract cancer (BTC), which arises from the biliary epithelium of the intrahepatic, extrahepatic, and gallbladder bile ducts, accounts for about 3% of all gastrointestinal cancers [1] and is the sixth leading cause of cancer death [2]. In Japan, perihilar bile duct, distal bile duct, and gallbladder cancers have overall 5-year survival rates of 24.2%, 39.1%, and 39.8%, respectively [3]. To date, surgery has been the exclusive curative therapy for BTC. In addition, survival post-surgery is short in cases involving positive resection margins, perineural invasion, lymph node metastasis, and undifferentiated adenocarcinoma in resected tissues [4].

To avoid unnecessary invasive procedures and determine the appropriate therapy, a precise diagnosis of tumor spread is important. Currently, more extensive surgery made possible by accumulated experience and recent technical advances has allowed for complete resection of BTC lesions (e.g., hepatopancreatoduodenectomy) [5]. By conducting a thorough examination pre-surgery, a positive resected margin is avoidable, improving risk factors related to postoperative survival. Earlier studies have reported the primary tumor’s superficial spread or extension in 31.6–39.3% of BTC cases, with more than 20-mm length of superficial spread in 14.6–17.9% cases [6,7]. These lesions can be identified using ultrasonography [8], multi detector-row computed tomography (MDCT) [9], and magnetic resonance imaging [10]. Intraductal ultrasonography (IDUS) during endoscopic retrograde cholangiopancreatography (ERCP) has been shown to be beneficial for both qualitative diagnosis and the diagnosis of the main tumor’s superficial spread [11,12]. However, these methods have a limited diagnostic accuracy in terms of the superficial tumor spread. In contrast, superficial spread of tumor can be diagnosed using peroral cholangioscopy (POCS), which has the advantage of allowing direct visualization of the bile duct lumen [13]. The diagnostic accuracy has been reported to increase when POCS is used with bile duct biopsy [14,15,16]. Furthermore, recent improvements in image resolution and the development of narrow-band imaging have enabled the detailed observation of surface structures and the fine vasculature of bile ducts [17]. Several POCS studies, focusing on surface and vessel structures, have reported on benign and malignant bile ducts findings [16,18,19,20]. However, to date, there are no reports systematically classifying POCS findings.

Recent advances in next-generation sequencing (NGS) have enabled rapid and comprehensive gene sequencing, which have allowed the identification of gene alterations in numerous tumors, including BTC [21,22]. Of note, targeted deep sequencing has a high sensitivity in detecting multiple gene mutations. The variant allele frequency (VAF) of genes reflect the fraction of tumor cells per sample and can be used to determine the tumor grade [23].

In this study, we developed a classification system based on POCS findings in surface and vessel structures to diagnose BTC tumor spread. The validity of this classification system was evaluated using histological diagnosis and gene mutation analysis in biopsy specimens. Furthermore, we examined the effectiveness of this classification in determining the extent of resection.

## 2. Results

### 2.1. Patient Characteristics and Assessment of Biopsied Samples

A total of 11 patients (8 men and 3 women) were enrolled in this study, with their median age being 70 (range, 59–79) years. Lesions were in the following regions: intrahepatic bile duct (*n* = 1), perihilar bile duct (*n* = 2), and distal bile duct (*n* = 8). Macroscopic classification revealed 5, 4, and 2 cases of papillary type, nodular type, and flat type, respectively. Histological tumor invasion around the bile duct (pT) and histological lymph node metastasis (pN) were evaluated according to the TNM Classification of Malignant Tumors (7th edition) [24]. Staging revealed 4 early-stage (<pT3) cases and 7 more advanced cases (≥pT3), with 7 pN0 cases and 4 pN1 cases (Table 1). Endoscopic procedures resulted in no complications. Surgical resection after POCS examination was performed in all patients.

The median amount of DNA extracted from 18 biopsied samples was 13.0 (range, 2.5–34.0) ng. However, the amount of DNA extracted from the other 12 samples was below the detection sensitivity. The median sequence read depth was 7570 (range, 261–16,055) (Table S1). Specimens of 12, 8, 2, and 8 parts in the bile ducts were biopsied from areas with F1, F2, F3, and F4, respectively. Specimens of 15, 5, and 10 were from areas with V1, V2, and V3, respectively.

### 2.2. Association between the F–V Classification and the Histological Assessment of Biopsied Samples

**Figure 1:**
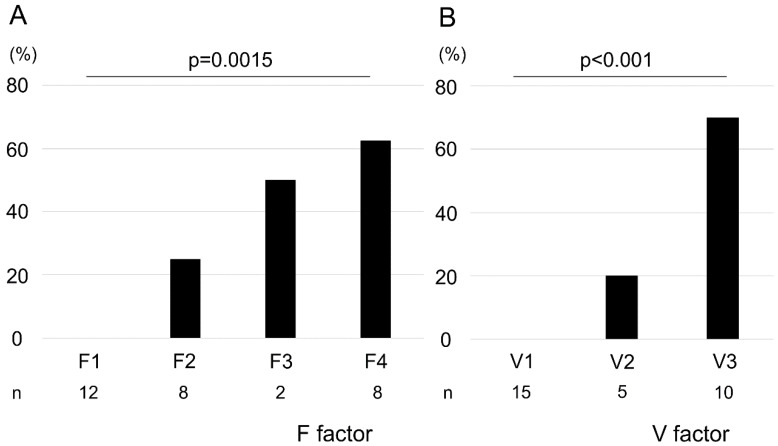
Association between pathological malignancy rate and F–V factors. Pathological malignancy rates increased with increasing F- and V-factor scores (**A**, F factor; **B**, V-factor). Statistical significance was determined using the Cochran–Armitage trend test.

We observed a positive correlation between F factor and V-factor scores (correlation coefficient: 0.91; Figure S1). The pathological malignancy rates with respect to the F factor were as follows: F1, 0%; F2, 25%; F3, 50%; and F4, 62.5% (Figure 1A). Similarly, those with respect to the V-factor were as follows: V1, 0%; V2, 20%; and V3, 70% (Figure 1B). We found that higher F–V scores significantly corresponded with higher histological malignancies of the biopsied specimens (F factor, *p* = 0.0015; V-factor, *p* < 0.001). However, no malignancy of the biopsied specimens was observed in POCS findings in terms of F1V1 and F2V1. Surgical margins were negative in 9 of 11 cases, and all stumps of these 9 cases were F1V1 in POCS findings. In 2 cases, surgical margins were positive with carcinoma in situ, and these cases had a positive surgical margin with F2V3 in POCS findings.

### 2.3. Association between the F–V Classification and Genetic Mutations

**Figure 2:**
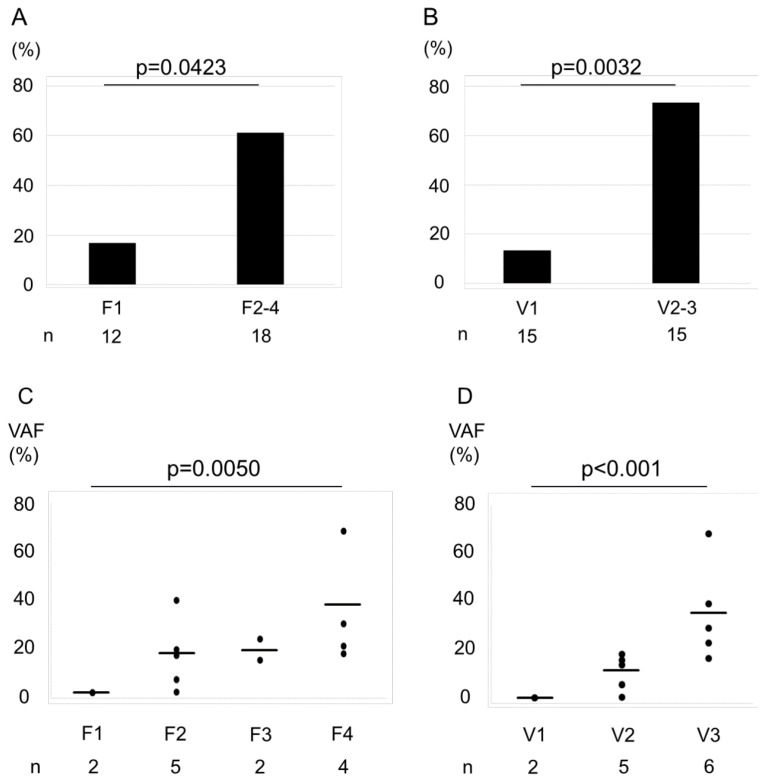
Association between the percentage of cases with a gene mutation and F–V-factor scores. Bar graphs represent the percentage of cases with a gene mutation by F factor score (**A**) and V-factor score (**B**). The rate of genetic mutations of F2–F4 was higher than F1 (A). The rate of genetic mutations of V2–V3 was higher than V1 (**B**). Statistical significance was assessed using χ^2^ test. The variant allele frequencies (VAFs) increased with increasing F- and V-factor scores (**C**, F factor; **D**, V-factor). Horizontal bars indicate the mean. Increasing trends were analyzed using the Jonckheere–Terpstra trend test. VAFs were plotted only for specimens with a confirmed gene mutation.

In the present study, of the 50 cancer-related genes that were examined, *TP53* (36%), *RB1* (27%), and *KIT* (18%) were the most frequently mutated ones. Of the tested samples, 13 (43.3%) had at least one gene mutation (Figure S2). The fraction of samples with a gene mutation according to the F factor was as follows: F1, 16.7%; and F2–F4, 61.1% (Figure 2A), whereas those with a mutation according to the V-factor were as follows: V1, 13.3%; V2–V3, 73.3% (Figure 2B). We observed that the differences between V-factor categories were statistically significant (F factor, *p* = 0.0423; V-factor, *p* = 0.0032). Furthermore, we found an increase in VAF of the mutated genes with increasing F- and V-factor scores (Figure 2C,D, *p* = 0.005 and <0.001, respectively).

### 2.4. Association between the Histological Assessment and Genetic Mutations in F–V Classification

**Figure 3:**
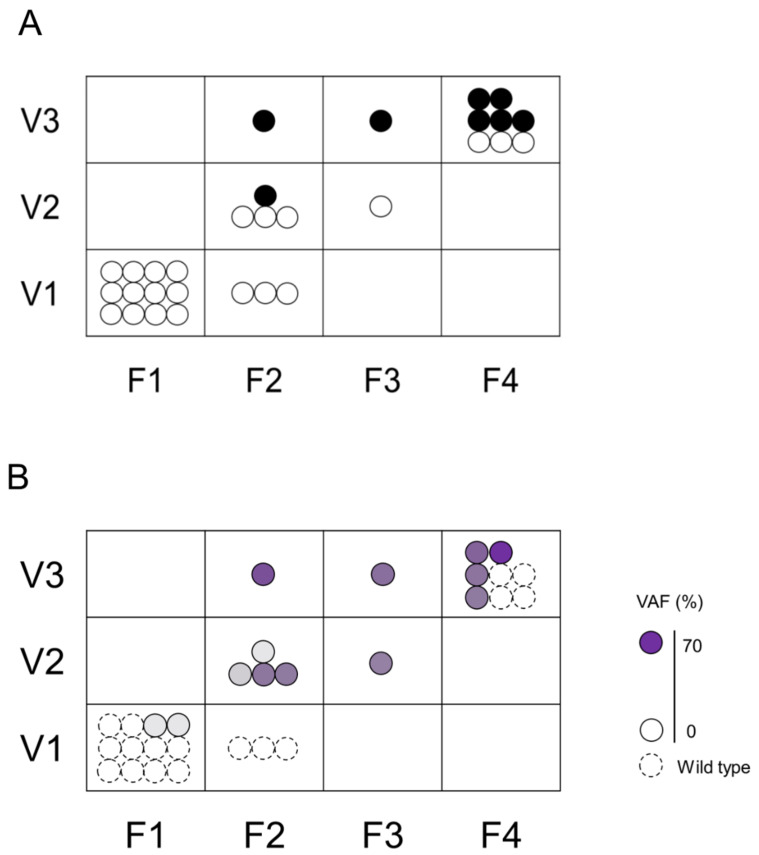
Association between the histological assessment and genetic mutations in F–V classification. The upper Figure (**A**) shows the association between F–V classification and histological assessment. Black circle (●) indicates malignancy and white circle (〇) indicates non-malignant lesions. The lower Figure (**B**) shows the association between F–V classification and genetic mutation. Dotted circles represent specimens without genetic mutation, and solid circles represent specimens with genetic mutation. The color density in the circle indicates the VAF.

We assessed the association between F–V classification and histological diagnosis (Figure 3A) or VAFs (Figure 3B) of biopsied specimens. The group evaluated as F1V1 or F2V1 in POCS were all histologically benign, had a low rate of genetic mutation, and a low gene mutation VAF. On the contrary, the groups evaluated as F4V3 or F3V3 or F2V3 in POCS were histologically malignant, had a high rate of genetic mutation, and had a high gene mutation VAF. The group evaluated as F3V2 or F2V2 in POCS were histologically benign, had a high rate of genetic mutation, and had a low gene mutation VAF.

**Figure 4:**
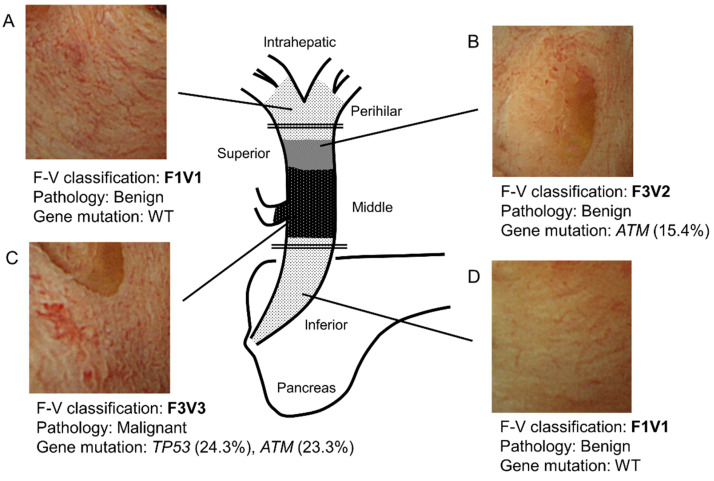
Schema of a representative bile duct carcinoma case. (**A**) In the perihilar bile duct, POCS showed the presence of a flat bile duct epithelium with a network of thin vessels (F1V1). In this region’s biopsied specimens, neither a tumor nor a genetic mutation was identified. (**B**) In the superior bile duct, POCS revealed a papillary bile duct epithelium with irregular, non-dilated vessels (F3V2). In this region’s biopsied specimens, no tumor was observed. However, a genetic mutation in *ATM* was found (VAF; 15.4%). (**C**) In the main lesion of the middle bile duct, POCS demonstrated the presence of a papillary bile duct epithelium with an irregular, dilated, and tortuous vessel (F3V3). The biopsied specimens showed adenocarcinoma. Genetic mutations in *TP53* (VAF, 24.3%) and *ATM* (VAF, 23.3%) were identified. (**D**) In the inferior bile duct, POCS revealed a flat bile duct epithelium, with a network of thin vessels (F1V1). In this region’s biopsied specimens, neither a tumor nor genetic mutation was observed. VAF, variant allele frequency; WT, wild type. The double line shows the resection line.

Figure 4 shows a representative case. Specifically, this case highlights the relationship among F–V classification of POCS findings, histology, and genetic mutations. In this case, the main tumor lies in the middle bile duct. It is classified as F3V3 according to POCS findings, malignant pathology, and gene mutations (Figure 4C). In addition, the perihilar and inferior bile duct has a benign lesion. The benign lesion in the inferior bile duct is classified as F1V1 according to POCS findings, with no gene mutation (Figure 4A,D). The tumor extends into the superior bile duct, categorized as F3V2 with a gene mutation, with a benign pathology (Figure 4B). These findings were consistent with those of the resected tissues that were pathologically diagnosed.

### 2.5. Association between F–V Classification and Pathology Diagnosis of Resected Stump

In total, 11 patients underwent the following procedures: pancreatoduodenectomy (*n* = 7), hepatectomy (*n* = 3), and extrahepatic bile tract resection (*n* = 1, Table 2). Of the 15 resected stumps, 13 were F1V1 and 2 were F2V3 according to the F–V classification. Histologically, the F1V1 stumps had no carcinoma and the F2V3 stumps had carcinoma in situ.

## 3. Discussion

In the present study, we classified the POCS findings of BTC cases based on the form of the bile duct surface (F factor) and vascular structures (V-factor). This new system is called “the F–V classification of POCS findings.” The system was validated by comparing it to the histological diagnosis and genetic mutation analysis in simultaneously biopsied specimens. Comparison with the histological diagnosis revealed a statistically significant increase of the malignancy rate with increasing F- and V-factor scores. Comparison with the mutation status showed an increased frequency of mutant variants in samples with an increase in the F- and V-factor scores. In addition, the F–V classifications of resected margins according to POCS findings were all accurate.

F–V classification is the first reported system to quantify and classify BTC based on POCS findings. Several reports have quantified POCS findings according to bile duct malignancies [16,19,20,25]. However, none of these have reported methods for stratification according to malignancy. We found that this approach is confusing when applied to diagnosis. On the contrary, we noticed that the reported observations of POCS findings could be categorized into 2 groups. The first group comprised surface structures of the bile duct such as “irregular fine granular pattern” [16], “irregular papillogranular surface” [19], “nodular elevated surface-like submucosal tumor” [19], “irregular surface or papillary projections” [20], and “luminal narrowing that was continuous with the main cancerous lesion” [20]. The second group comprised vascular structures such as “fish-egg-like appearance” [16,19], “irregularly dilated and tortuous vessels” [20], and “irregular or spider vascularity” [25]. Therefore, we decided to develop a classification system by further scoring these POCS findings according to the degree of malignancy. Specifically, our F–V classification of POCS findings is based on these systematic studies. Its validity was verified by comparing it with histological diagnosis and genetic mutation analysis in biopsied specimens.

Recent advances in NGS have enabled the identification of comprehensive gene profiles of numerous cancers, including BTC, which has been reported to have frequent alterations in *TP53*, *KRAS*, *SMAD4*, and *BAP1* genes [22]. Characteristic gene alterations vary depending on the main tumor site. For example, alterations in *TP53*, *KRAS*, *BAP1*, *ARID1A*, *IDH,* and *SMAD4* are generally observed in intrahepatic cholangiocarcinoma, whereas *TP53*, *KRAS*, *SMAD4*, and *ERBB2* mutations are associated with extrahepatic cholangiocarcinoma [22,26]. *TP53* alterations are characteristic of extrahepatic cholangiocarcinoma. In our study, *TP53* mutations were the most frequently observed one in extrahepatic cholangiocarcinoma. For other mutations, we did not observe the same tendency as reported. We believe that this discrepancy may be because of the small sample size in our study, not accurately reflecting distribution of gene alterations. The VAF, also known as the mutant allele frequency, indicates tumor cellularity from extracted DNA. The VAF has been used to predict the degree of malignancy [23] and the reactivity to drugs [27] in certain tumors. Thus, we used the VAFs of biopsied bile duct specimens to classify the degree of malignancy. We found a correlation between the fraction of cases with a mutation and the F–V classification of POCS findings. The same was true for the histological diagnosis.

To select the appropriate surgical procedure, the superficial spread of a tumor should be precisely diagnosed by POCS. This is because BTC is often accompanied by superficial spread in the bile duct [6,7]. Postoperative 5-year survival rate is unaffected by positive surgical margins with carcinoma in situ [6,28]. However, because positive margins are reported to affect longer post-surgical survival, we should aim for negative surgical margins [29]. On the contrary, more extensive biliary resection may greatly increase surgical stress. Specifically, resection of the upstream bile duct requires hepatectomy, whereas the resection of the downstream bile duct requires pancreatectomy. These expanded surgical procedures are associated with the risk of surgery-related death [30]. Thus, an adequate surgery, neither excessive nor insufficient, should be chosen based on the disease extent, patient’s general condition, and the imposed surgical risks. Currently, the final resection margin in BTC surgery is determined by intraoperative frozen-section diagnosis, which is not always correct [31,32]. Reports show that the epithelial layer’s correct diagnosis rate is considerably lower than subepithelial layer [31]. POCS can directly visualize the bile duct lumen with biopsy, thereby aiding the diagnosis of BTC’s spread [18,20]. Here, assuming that F2V3 in F–V classification is malignant, the correct diagnosis rate of the F–V classification in stump evaluation was 100%. Therefore, we believe that the F–V classification may be more effective in the diagnosis of BTC superficial spread versus intraoperative frozen sections. Furthermore, in the future, it may become possible to determine the range of resection by POCS findings and genetic variation of the biopsy specimen. In summary, the F–V classification of POCS findings, together with intraoperative frozen-section diagnosis, may enable the precise diagnosis of a surgical stump.

Multiple clinical implications were fostered by the findings of this study. First, the F–V classification of POCS findings may guide in the assessment of the potential risks in diagnosed bile duct tumors. In a prospective multicenter study, the diagnostic accuracy of BTC superficial spread has been reported to be 83.7% (41/49) for POCS findings and 92.9% (39/42) for POCS with biopsy [19]. However, even with the addition of biopsy to the POCS diagnosis, the accuracy remains to be imperfect, probably because biopsy specimens were too small for histological diagnosis and the gray zones of the histologic characteristics that exist precluded differentiation between benignity and malignancy. As shown in Figure 4, some samples with mutations but without histological confirmation of malignancy had V2 findings by the F–V classification. In other words, a V2 finding in the F–V classification may be equivalent to a histologic diagnosis of malignancy or to a potential risk of malignancy because only this finding can allow the identification of mutated bile duct epithelium without histological confirmation of malignancy. Furthermore, in another study, we recently reported similar concepts about the relation between the endoscopic findings of colorectal tumors and genetic abnormalities [33]. Accumulated gene alterations in the adenoma components of colorectal carcinoma could be diagnosed based on irregular surface pattern findings on magnifying endoscopy. this means that a gray zone with accumulated genetic changes exists that cannot be diagnosed as malignant tumor via histology. Second, in accordance with the preceding discussion, we believe that the F–V classification may be useful in determining whether the surgical margins for the papillary and nodular expanding types of BTC are distal or perihilar. The papillary and nodular expanding types of BTC tend to show extensive spread on histology [6,28,34] and often require hepatectomy, in addition to pancreatoduodenectomy [6]. The F–V classification of POCS findings may be beneficial, especially for the gray zone that cannot be diagnosed even by biopsy.

There are several limitations in our study. First, this was a single-centered retrospective study with a small sample size. Although 36 patients underwent resection, 22 of them underwent POCS during the study period and only 11 patients who had available POCS findings and mutational analysis of the biopsied samples were included in our study. Second, no correlation was found between F-factors and mutation frequency in tissue samples, which might be because of insufficient sampling, especially of the main lesions. This could have likely reduced the chances of detecting target gene mutations. Thus, the rate of malignancy in histological diagnosis and that of genetic mutation are not high in BTC main lesions. Alternatively, different gene mutations other than those analyzed in this study might have existed in our biopsied samples. Third, there are inflammatory biliary diseases such as IgG4-related sclerosing cholangitis, which should be differentiated from BTC. We did not assess whether our POCS classification would be useful for the diagnosis of such inflammatory biliary diseases. Therefore, future prospective studies with a large sample size and the selection of more appropriate target genes may improve the correlation between the POCS findings and analysis of the biopsied specimens.

In conclusion, we classified POCS findings of BTC as “the classification of POCS findings.” In addition, we evaluated its validity by performing histological diagnosis and genetic mutation analysis on biopsied specimens. Although the findings of this pilot study need further verification, we hope that this classification would help stratify the grade of malignancy around the tumor lesion and enable the selection of an appropriate surgical procedure by precisely diagnosing the superficial spread of BTC tumors.

## 4. Materials and Methods

### 4.1. Patients and Samples

We retrospectively reviewed the medical records of 11 patients who underwent POCS examination to diagnose BTC and its superficial spread before surgery at Yamanashi University Hospital between January 2013 and December 2017. We assessed 2 to 5 regions up- and downstream of the main lesion (Figure 4) and took 2–3 biopsies from each region of the bile duct using POCS, which yielded a total of 70 specimens from 11 patients. Of these specimens, 30 good quality specimens were included in the study (Table S2). The remaining 40 samples were excluded because of inability to extract DNA (*n* = 3), poor quality of the extracted DNA (*n* = 10), or duplication of collection sites (*n* = 27). When several samples were obtained from the same region, we chose those with the best size, quantity, and quality of the extracted DNA. The Human Ethics Review Committee of Yamanashi University Hospital approved this study (Receipt number: 1523, From January 2017 to March 2019).

### 4.2. Bile Duct Biopsies Using POCS

A video cholangioscope (CHF-B260, Olympus Medical Systems, Tokyo, Japan) with outer diameters of 3.4 and 1.2 mm was used as the baby scope. It was passed through the side-viewing mother scope (TJF-240, Olympus Medical Systems) with a 4.2-mm working channel into the bile duct using a 0.025-inch guide wire. Before inserting the baby scope into the bile duct, endoscopic sphincterotomy, or endoscopic papillary balloon dilation was performed. The bile duct was irrigated with sterile saline solution during the POCS procedure through a working channel. Furthermore, the bile duct surface was observed. Tissues were sampled according to bile duct assessment using thin biopsy forceps (Spybite Biopsy Forceps, Boston Scientific, Marlborough, MA, USA) (Figure S3). A pathologist performed histological diagnosis on hematoxylin-eosin-stained slides. Malignancy was noted as suspicious or definite. Patients were under conscious sedation using intravenous flunitrazepam (5–10 mg) during all endoscopic procedures, and all 11 cases in this study underwent ERCP with the introduction of a plastic stent or an endoscopic nasobiliary drainage tube, days before POCS. No cases of cholangitis were observed when performing POCS.

### 4.3. Form-Vessel Classification of Bile Duct Carcinoma POCS Findings and Its Diagnostic Accuracy of Surgical Margins

**Figure 5:**
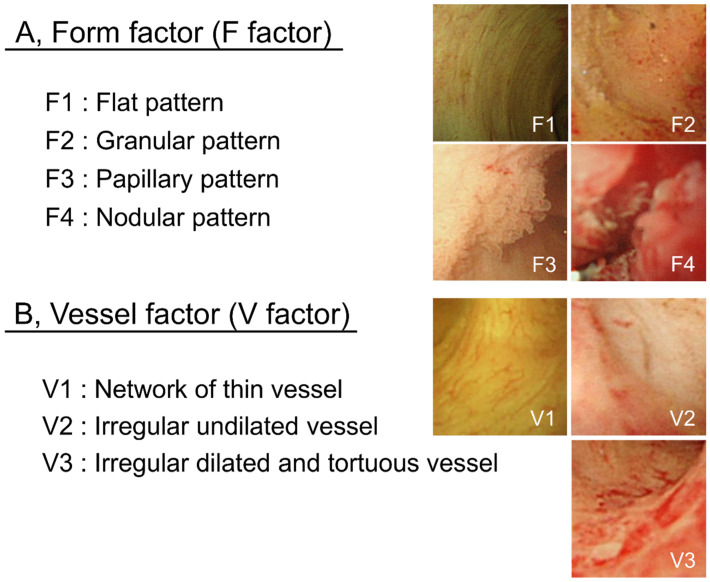
F–V classification of bile duct POCS findings. Bile duct epithelium POCS findings were classified into 4 surface structure groups (F1–F4) (**A**) and 3 vessel pattern groups (V1–V3) (**B**).

POCS findings of bile duct surface were evaluated according to the form of the bile duct surface (F factor) and vessel structure (V-factor) in the following regions: left and right hepatic duct, the confluence of the hepatic ducts, and the superior, middle, and inferior parts of the bile duct lumen in order to determine whether pancreatoduodenectomy or hepatectomy was required. We used 4 grades to classify the bile duct surface form: F1, flat pattern; F2, granular pattern; F3, papillary pattern; and F4, nodular pattern. Vessel structures were classified into 3 grades: V1, network of thin vessels; V2, irregular non-dilated vessel; and V3, irregular dilated and tortuous vessels (Figure 5). Atypicality was worse and more severe with a higher score. This classification system was named “the F–V classification of POCS findings.” At least 3 gastroenterologists specialized in the bile ducts evaluated the classified findings.

In additionally, we evaluated whether the POCS findings could accurately diagnose the pathology of resected margins. We correlated the POCS findings with the site of resection by measuring the distance from the boundary line of bile duct carcinoma to the point of confirmation, such as the junction of the cystic duct and the confluence of the hepatic ducts, on both POCS examination and surgery. Moreover, the pathology of surgical stumps was evaluated on the frozen section during surgery and after resection using formalin-fixed paraffin-embedded tissues. There were 15 resected stumps in 11 cases. All of them had liver side stump. Moreover, 4 patients who had undergone hepatectomy or extrahepatic bile tract resection had duodenal side stump. The histology of resected stumps was compared with preoperative assessment by the POCS findings.

### 4.4. Genetic Mutational Analysis of Biopsied Specimens

DNA extraction and mutational analysis of biopsied specimens were performed as previously reported [35]. Briefly, biopsied specimens were laser-capture microdissected using the ArcturusXT Laser-Capture Microdissection System (Life Technologies, Carlsbad, CA, USA). Tissue was obtained from 8-μm thick sections of formalin-fixed paraffin-embedded (FFPE) samples. DNA was extracted using the GeneRead DNA FFPE Kit (QIAGEN, Hilden, Germany), following the manufacturer’s instructions. Extracted DNA quantity and quality were assessed using NanoDrop (Thermo Fisher, Waltham, MA, USA) and Qubit (Thermo Fisher) platforms. Extracted DNA (10 ng) was amplified using barcode adaptors (Ion Xpress Barcode Adapters 1-96 Kit, Life Technologies) by the Ion AmpliSeq Cancer Horspot panel v.2 (Thermo Fisher), which contains 207 primer pairs and targets approximately 2800 hotspot mutations located in the following 50 cancer-related genes: *ABL1*, *AKT1*, *ALK*, *APC*, *ATM*, *BRAF*, *CDH1*, *CDKN2A*, *CSF1R*, *CTNNB1*, *EGFR*, *ERBB2*, *ERBB4*, *EZH2*, *FBXW7*, *FGFR1*, *FGFR2*, *FGFR3*, *FLT3*, *GNA11*, *GNAS*, *GNAQ*, *HNF1A*, *HRAS*, *IDH1*, *JAK2*, *JAK3*, *IDH2*, *KDR/VEGFR2*, *KIT*, *KRAS*, *MET*, *MLH1*, *MPL*, *NOTCH1*, *NPM1*, *NRAS*, *PDGFRA*, *PIK3CA*, *PTEN*, *PTPN11*, *RB1*, *RET*, *SMAD4*, *SMARCB1*, *SMO*, *SRC*, *STK11*, *TP53*, and *VHL*. Such genes are available in the COSMIC database [36]. The barcoded libraries were amplified by emulsion PCR on Ion Sphere particles. Sequencing was then performed on an Ion Chef System and an Ion Proton Sequencer (Life Technologies) using the Ion PI Hi-Q Chef Kit (Life Technologies), based on the manufacturer’s instructions. Variants were identified using Ion reporter software version 5.10 (Thermo Fisher), and those with a VAF > 2% (with a sequence read depth >100) were considered true variants. The highest VAF among several mutated genes in the same sample was used as the respective sample’s VAF.

### 4.5. Statistical Analysis

Statistical analysis was performed to validate our classification system. Specifically, we used the Cochran–Armitage trend test to determine the rates of histological malignancy and gene mutation. The Jonckheere–Terpstra trend test was used to compare the VAFs between F and V factors. A *P* value of <0.05 was considered statistically significant. All statistical analyses of recorded data were performed using the Excel statistical software package (Ekuseru–Toukei 2012; Social Survey Research Information Co., Ltd., Tokyo, Japan).

## Figures and Tables

**Table 1:** Baseline characteristics of patients.

Characteristics	Values (*n* = 11)
**Age, mean ± SD (years)**	**69.9 ± 6.9**
Sex (*n*)	
Male	8
Female	3
Location of main lesions in CBD (*n*)	
Bh	1
Bp	2
Bd	8
Macroscopic tumor type (*n*)	
Papillary	5
Nodular	4
Infiltrating	2
Histology (*n*)	
Well	5
Moderate	6
Depth of invasion (*n*)	
<pT3	4
≥pT3	7
Lymph node metastasis (*n*)	
pN0	7
pN1	4

CBD, common bile duct; Bh, intrahepatic bile duct; Bp, perihilar bile duct; Bd, distal bile duct.

**Table 2:** Relationship between F–V classification and pathology diagnosis of resected stump.

Case	Operation	Findings of Ductal Margin (Duodenum Side)		Findings of Ductal Margin (Liver Side)	
		F–V Classification	Pathological Diagnosis	F–V Classification	Pathological Diagnosis
1	PD	-	-	F2V3	CIS
2	Hepatectomy	F2V3	CIS	F1V1	negative
3	PD	-	-	F1V1	negative
4	*	F1V1	negative	F1V1	negative
5	Hepatectomy	F1V1	negative	F1V1	negative
6	PD	-	-	F1V1	negative
7	PD	-	-	F1V1	negative
8	Hepatectomy	F1V1	negative	F1V1	negative
9	PD	-	-	F1V1	negative
10	PD	-	-	F1V1	negative
11	PD	-	-	F1V1	negative

*, Extrahepatic bile tract resection; PD, Pancreatoduodenectomy; CIS, Carcinoma in situ.

## Supplementary Materials

Supplementary materials can be found at https://www.mdpi.com/1422-0067/21/9/3311/s1.
