# Supplementary material for: Form-Vessel Classification of Cholangioscopy Findings to Diagnose Biliary Tract Carcinoma’s Superficial Spread
Source: Int J Mol Sci. 2020 May 7;21(9):3311. doi: 10.3390/ijms21093311 (PMC7246671; doi:10.3390/ijms21093311)
Supplement: Supplementary file 1 [file ijms-21-03311-s001.pdf]

# Form-Vessel Classification of Cholangioscopy Findings to Diagnose Biliary Tract Carcinoma's Superficial Spread

**Yoshimitsu Fukasawa, Shinichi Takano, Mitsuharu Fukasawa, Shinya Maekawa, Makoto Kadokura, Hiroko Shindo, Ei Takahashi, Sumio Hirose, Satoshi Kawakami, Hiroshi Hayakawa, Tatsuya Yamaguchi, Yasuhiro Nakayama, Taisuke Inoue, Tadashi Sato and Nobuyuki Enomoto**

First Department of Internal Medicine, Faculty of Medicine, University of Yamanashi, Chuo, Yamanashi, Japan

**Supplementary Material**

| | | | | |
|---|---|---|---|---|
| V3 | | 1 | 1 | 8 |
| V2 | | 4 | 1 | |
| V1 | 12 | 3 | | |
| | F1 | F2 | F3 | F4 |

**Figure S1.** Association between pathology and F–V factors. The 30 biopsy specimens are summarized in the Table in which F and V factors are combined. The numbers in the squares represent the number of biopsied specimens in each F–V evaluations. A positive correlation was observed between F- and V-factor scores (correlation coefficient: 0.91).

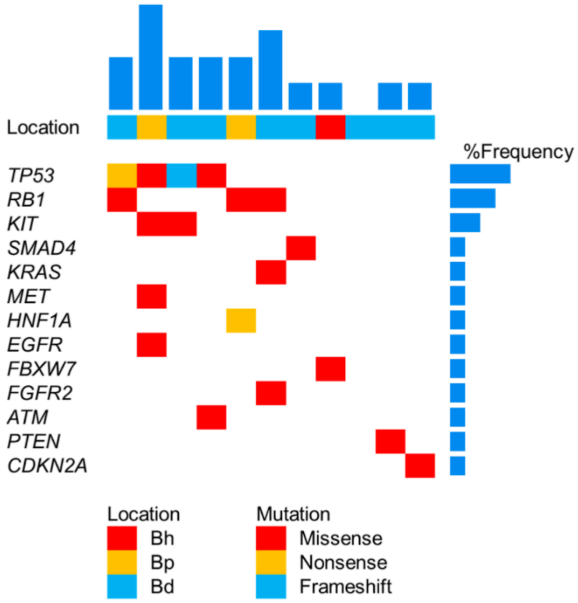


**Figure S2.** Genetic mutations detected in biopsied samples of the main tumor. The upper panel shows the tumor location and the number of mutations in each case as a bar graph. The squares in the main panel show the mutations detected in the tumor samples divided by mutation type. The histogram on the right shows the percentage of cases with a mutation in each gene, detected by screening for 50 cancer-related genes. Bh, intrahepatic bile duct; Bp, perihilar bile duct; Bd, distal bile duct.

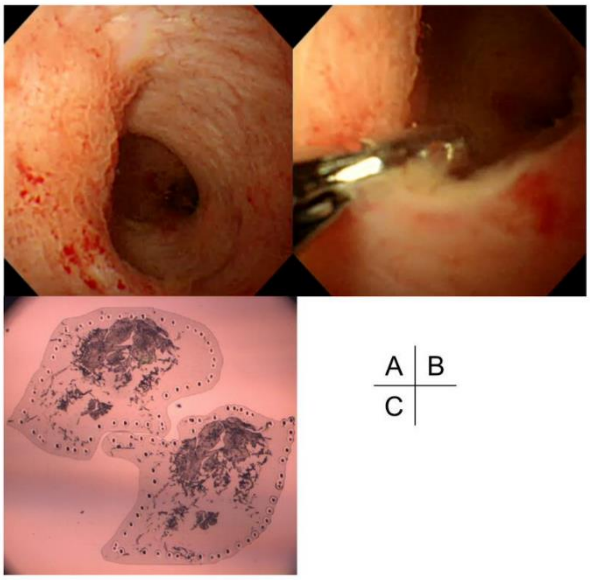


**Figure S3.** Bile duct biopsies conducted via peroral cholangioscope (POCS)**.** (**A**) POCS findings of the bile duct were determined as F2V3. (**B**) Bile duct epithelium with F2V3 findings biopsied via POCS. (**C**) Formalin-fixed, paraffin-embedded biopsied specimens were laser-capture microdissected and used for DNA extraction.

**Table S1.** DNA status of all biopsy specimens.

| Number | Case | DNA extracted (ng) | Mean read depth |
|---|---|---|---|
| 1 | 1 | 23.2 | 4025 |
| 2 | 2 | < 2.0 | 6835 |
| 3 | 2 | < 2.0 | 3697 |
| 4 | 3 | < 2.0 | 9630 |
| 5 | 3 | < 2.0 | 4364 |
| 6 | 3 | < 2.0 | 8974 |
| 7 | 3 | 25.8 | 2298 |
| 8 | 4 | 13 | 7500 |
| 9 | 4 | 26 | 4166 |
| 10 | 4 | 7.9 | 7759 |
| 11 | 4 | 14.1 | 12761 |
| 12 | 5 | 4.6 | 261 |
| 13 | 6 | 34 | 7640 |
| 14 | 6 | 2.7 | 2322 |
| 15 | 6 | < 2.0 | 5721 |
| 16 | 6 | 7.2 | 12055 |
| 17 | 7 | < 2.0 | 11024 |
| 18 | 7 | 14.8 | 9870 |
| 19 | 7 | 13 | 6738 |
| 20 | 7 | 29.6 | 1996 |
| 21 | 8 | < 2.0 | 8018 |
| 22 | 9 | < 2.0 | 13359 |
| 23 | 9 | 3.8 | 9232 |
| 24 | 9 | 22.4 | 6671 |
| 25 | 10 | < 2.0 | 16055 |
| 26 | 10 | < 2.0 | 12821 |
| 27 | 11 | < 2.0 | 12766 |
| 28 | 11 | 2.5 | 12480 |
| 29 | 11 | 7.4 | 6325 |
| 30 | 11 | 7.7 | 3666 |

**Table S2.** Evaluation of F–V classification and genetic mutation for each biopsy specimen.

| Number | Case | Main-tumor location | F factor | V factor | Pathology | Genes with mutation (VAF, %) |
|---|---|---|---|---|---|---|
| 1 | 1 | Bd | 4 | 3 | malignant | *TP53* (68.7), *RB1* (17.7) |
| 2 | 2 | Bp | 2 | 3 | malignant | *TP53* (40.2), *KIT* (7.8), *MET* (4.7), *EGFR* (3.8) |
| 3 | 2 | Bp | 1 | 1 | benign | |
| 4 | 3 | Bd | 2 | 2 | malignant | *KIT* (2.4), *TP53* (2.2) |
| 5 | 3 | Bd | 2 | 2 | benign | *TP53* (7.5) |
| 6 | 3 | Bd | 1 | 1 | benign | |
| 7 | 3 | Bd | 4 | 3 | malignant | |
| 8 | 4 | Bd | 3 | 2 | benign | *ATM* (15.4) |
| 9 | 4 | Bd | 3 | 3 | malignant | *TP53* (24.3), *ATM* (23.3) |
| 10 | 4 | Bd | 1 | 1 | benign | |
| 11 | 4 | Bd | 1 | 1 | benign | |
| 12 | 5 | Bp | 4 | 3 | malignant | *HNF1A* (18.1), *RB1* (12.7) |
| 13 | 6 | Bd | 2 | 2 | benign | *RB1* (17.4), *KRAS* (5.3), *FGFR2* (4.7) |
| 14 | 6 | Bd | 2 | 2 | benign | *RB1* (19.8), *FGFR2* (4.0) |
| 15 | 6 | Bd | 1 | 1 | benign | |
| 16 | 6 | Bd | 4 | 3 | benign | |
| 17 | 7 | Bd | 4 | 3 | malignant | *SMAD4* (30.4) |
| 18 | 7 | Bd | 1 | 1 | benign | |
| 19 | 7 | Bd | 2 | 1 | benign | |
| 20 | 7 | Bd | 2 | 1 | benign | |
| 21 | 8 | Bh | 4 | 3 | benign | *FBXW7* (21.4) |
| 22 | 9 | Bd | 1 | 1 | benign | |
| 23 | 9 | Bd | 2 | 1 | benign | |
| 24 | 9 | Bd | 4 | 3 | malignant | |
| 25 | 10 | Bd | 1 | 1 | benign | *PTEN* (2.2) |
| 26 | 10 | Bd | 1 | 1 | benign | |
| 27 | 11 | Bd | 1 | 1 | benign | *CDKN2A* (2.2) |
| 28 | 11 | Bd | 1 | 1 | benign | |
| 29 | 11 | Bd | 1 | 1 | benign | |
| 30 | 11 | Bd | 4 | 3 | benign | |
